# Digital Health Solutions for Cardiovascular Disease Prevention: Systematic Review

**DOI:** 10.2196/64981

**Published:** 2025-01-23

**Authors:** Yihan Qi, Emma Mohamad, Arina Anis Azlan, Chenglin Zhang, Yilian Ma, Anqi Wu

**Affiliations:** 1 Centre for Research in Media and Communication Faculty of Social Sciences and Humanities Universiti Kebangsaan Malaysia Selangor Malaysia; 2 Universiti Kebangsaan Malaysia Komunikasi Kesihatan (Healthcomm) - UKM Research Group Selangor Malaysia

**Keywords:** cardiovascular disease prevention, cardiovascular outcomes, digital technologies, remote care, mobile phone

## Abstract

**Background:**

Cardiovascular disease (CVD) is a major global health issue, with approximately 70% of cases linked to modifiable risk factors. Digital health solutions offer potential for CVD prevention; yet, their effectiveness in covering the full range of prevention strategies is uncertain.

**Objective:**

This study aimed to synthesize current literature on digital solutions for CVD prevention, identify the key components of effective digital interventions, and highlight critical research gaps to inform the development of sustainable strategies for CVD prevention.

**Methods:**

Following PRISMA (Preferred Reporting Items for Systematic Reviews and Meta-Analyses) guidelines, we conducted a comprehensive search in Web of Science, Scopus, and PubMed to identify original English-language studies published between January 2000 and May 2024 that examined primary or secondary CVD prevention through digital solutions. The exclusion criteria included: telephone-only interventions, abstract-only publications, methodology-focused studies without primary data, studies without participants or specific groups, and studies with no follow-up period. The literature search used the string with terms like “digital health,” “mHealth,” “mobile health,” “text message,” “short message service,” “SMS,” “prevention,” “prevent,” “cardiovascular disease,” “CVD,” etc. Study bias was assessed using the RoB 2 (Cochrane Collaboration) and the ROBINS-I tool (Cochrane Collaboration). Data on prevention components, prevention types, study design, population, intervention, follow-up duration, personnel, and delivery settings were extracted.

**Results:**

A total of 2871 studies were identified through the search. After excluding ineligible studies, 30 studies remained, including 24 randomized controlled trials. The reviewed digital solutions for CVD prevention focused on baseline assessment (29/30, 97%), physical activity counseling (18/30, 60%), tobacco cessation (14/30, 47%), blood pressure management (13/30, 43%), and medication adherence (10/30, 33%). The technologies used were categorized into 3 types, smartphones and wearables (16/30, 53%), email and SMS communications (12/30, 40%), and websites or web portals (3/30, 10%). The majority of the study outcomes addressed blood pressure (14/30, 47%), exercise capacity (12/30, 40%), weight (12/30, 40%), and lipid profile (11/30, 37%), while fewer focused on nicotine dependence (9/30, 30%), medication use (8/30, 27%), quality of life (7/30, 23%), dietary habits (5/30, 17%), intervention adherence (4/30, 13%), waist circumference (4/30, 13%), and blood glucose levels (2/30, 7%).

**Conclusions:**

Digital solutions can address challenges in traditional CVD prevention by improving preventive behaviors and monitoring health indicators. However, most evaluated interventions have focused on medication use, quality of life, dietary habits, adherence, and waist circumference. Further studies are needed to assess the long-term impact of more comprehensive interventions on key cardiovascular outcomes.

## Introduction

Cardiovascular disease (CVD) remains a significant worldwide health issue, making a contribution to global mortality. Approximately 70% of CVD cases are due to modifiable risk factors [1], these include lifestyle-related elements such as physical inactivity, poor dietary habits, high levels of blood pressure, and tobacco use. CVD prevention encompasses three stages: primary, secondary, and tertiary prevention. Primary prevention targets high-risk individuals without CVD [2], secondary prevention focuses on those with established CVD, and tertiary prevention is for individuals seriously affected by CVD and aims to enhance their life expectancy [3]. Despite the evidence supporting the effectiveness of CVD prevention initiatives, there is a significant difference between the potential benefits and the actual participation of individuals. Participation rates in preventive strategies still remain low, and adherence to lifestyle changes and medical recommendations is still unstable [4,5]. This arises from challenges such as socioeconomic inequalities, differences in the timing of participation, limited awareness or understanding of preventive strategies, restricted access to health care resources, and barriers related to transportation and distance [6]. Furthermore, barriers in health care systems, such as insufficient funding, fragmented care delivery, and inadequate integration, further hinder the adoption of CVD prevention measures [3].

Recent technological advances in CVD prevention offer solutions to the limitations of traditional facility-based measures. Mobile apps, wearable devices, telemedicine, and remote monitoring systems can improve individual engagement and adherence. These approaches can provide personalized instructions, real-time monitoring, and remote consultations, making it easier to manage their cardiovascular health. Moreover, they can reach broader populations, including those with limited access to health care facilities. This review employs digital health solutions to incorporate a range of technologies to facilitate care delivery. This way acknowledges the variety of digital tools available, including eHealth, mobile health (mHealth), SMS, wearable devices, mobile apps, and telemedicine.

Current studies have shown that digital solutions, such as mobile apps, hold promise for CVD prevention, but their effectiveness has mainly been demonstrated in limited settings, and broad implementation in clinical practice remains rare [7,8]. In addition, there is limited understanding of how comprehensively these digital solutions address the key components of CVD prevention and achieve targeted health outcomes. While these innovations hold the potential to enhance CVD prevention by improving accessibility, efficiency, and individual engagement, significant barriers remain. Challenges such as regulatory constraints, interoperability issues, and systemic limitations must be addressed to facilitate their effective integration into routine healthcare practices.

This study aims to evaluate the current landscape of digital technologies for CVD prevention, focusing on the comprehensiveness and effectiveness of digital solutions. Specifically, we serve multiple purposes, first, it integrates existing literature on digital solutions for CVD prevention; second, it identifies key components of CVD prevention that are effectively addressed through digital solutions; and third, it charts the gaps that need attention to facilitate the sustainable integration of digital solutions for CVD prevention into clinical practice.

## Methods

### Study Design

A systematic review was conducted following PRISMA (Preferred Reporting Items for Systematic Reviews and Meta-Analyses) guidelines [9] (Multimedia Appendix 1) to ensure transparency and consistency in reporting. This review aimed to answer two key research questions, that are (1) “What types of digital technologies are utilized in CVD prevention studies, including their sample sizes, intervention durations, follow-up periods, and primary findings?” and (2) “How comprehensive and effective are these CVD prevention solutions?”

### Eligibility Criteria

All studies published in English from January 2000 to May 2024 that examined digital solutions aimed at CVD prevention were considered for inclusion. Since Frank introduced the term “digital health” in the 2000s [10], it has transformed health care practices, bringing innovative approaches to CVD prevention worldwide. This review focused on the period from January 2000 to May 2024 (present) to capture the comprehensive applications of digital health technologies in CVD prevention, with an emphasis on primary and secondary prevention. In contrast, tertiary interventions, including coronary angioplasty, stenting, and bypass surgery, were excluded because their primary focus was on halting disease progression rather than prevention, which was beyond the objectives and scope of this study. In this review, the term “digital” refers to advanced technologies that facilitate remote, interactive, and personalized interventions. These technologies went beyond basic telephonic communication and included internet-based platforms, wearable devices that monitored and provided real-time physiological feedback, as well as mobile apps designed to track health behaviors, deliver educational content, and offer virtual guidance. As noted in the American College of Cardiology Scientific Statement on CVD Prevention [11], studies that only relied on telephone interventions were excluded. Specifically, studies were included if they (1) were original research using telemedicine or digital methods specifically targeting primary or secondary CVD prevention, (2) reported findings on feasibility and usability, and (3) were published in English. In contrast, studies were excluded if they (1) relied exclusively on telephone interventions, (2) were only available as abstracts without full-text access, (3) only focused on methodology without primary data, (4) did not involve participants or specific groups, or (5) lacked a follow-up period, such as cross-sectional studies without outcome tracking over time.

### Information Source

A comprehensive search was conducted for studies published between January 2000 and May 2024, using the Web of Science, Scopus, and PubMed databases for the selection process.

### Search Strategy

The search strategy used the PICO (Population, Intervention, Comparison, Outcome)-based search string, combining keywords and abbreviations related to digital health care technologies, prevention strategies, and CVD. Terms included “digital health,” “mHealth,” “mobile health,” “text message,” “short message service,” “SMS,” “prevention,” “prevent,” “cardiovascular disease,” “CVD,” etc. A comprehensive list of search terms was provided in Multimedia Appendix 2. 2 researchers (YQ and CZ) independently screened and identified relevant studies. Any discrepancies were resolved through discussion, with additional input from other researchers (EM and AAA) as needed to reach a consensus.

### Selection Process

To ensure reliability and remove duplicates, 2 researchers (YQ and CZ) independently imported the articles into EndNote 21 (Clarivate) and conducted the initial screening. Discrepancies in screening decisions were resolved by a third researcher (EM). Following title and abstract screening, 2 researchers (YQ and CZ) independently conducted a full-text review, with any discrepancies resolved through consultation with the third researcher (EM).

### Data Collection, Data Items, and Data Synthesis

Data collection was conducted by 2 researchers (YQ and CZ), and any disagreements were resolved through consultation with a third researcher (EM). Our approach follows the American Heart Association consensus statement [12] and Guide to Primary Prevention of Cardiovascular Diseases [13] about the components of primary and secondary CVD prevention. In addition, we divided the digital solutions outlined in each study into two groups: (1) those that operated independently and (2) those that enhanced traditional approaches to CVD prevention. According to Wongvibulsin et al [14], studies were classified as standalone solutions if the intervention primarily involved remote teaching, with the initial face-to-face meeting used for onboarding, baseline assessment, or outcome evaluation, provided that the primary intervention was delivered remotely. Moreover, we observed other characteristics including CVD prevention types, study countries, study designs applied, participation and population, duration of intervention or follow-up, personnel used, and delivery settings. Due to the significant heterogeneity among the publications, a quantitative synthesis and meta-analysis were not feasible. As a result, this study emphasized a qualitative synthesis, and data collection was conducted using Microsoft Excel 2023.

### Risk of Bias Assessment and Reporting Bias Assessment

The risk of bias for all included studies was independently assessed by 2 researchers (YQ and CZ), who also verified each other’s findings. Any disagreements were resolved through consultation with a third researcher (EM). Methodological rigor was ensured for each study type by the Cochrane Collaboration’s Risk of Bias (RoB) 2 tool [15] for randomized studies and the ROBINS-I tool [16] for nonrandomized studies. For randomized studies, we evaluated risks associated with the randomization process, deviations from intended interventions, missing outcome data, outcome measurement, and result selection. Each risk was classified as “low risk,” “some concerns,” or “high risk,” based on standardized criteria and expert judgment. For nonrandomized studies, we examined risks related to confounding bias, selection bias, intervention classification bias, deviations from intended interventions, missing data bias, outcome measurement bias, and reporting bias. Each risk was rated as “low,” “moderate,” “serious,” or “critical,” based on standardized criteria and expert judgment.

## Results

### Study Screening

**Figure 1:**
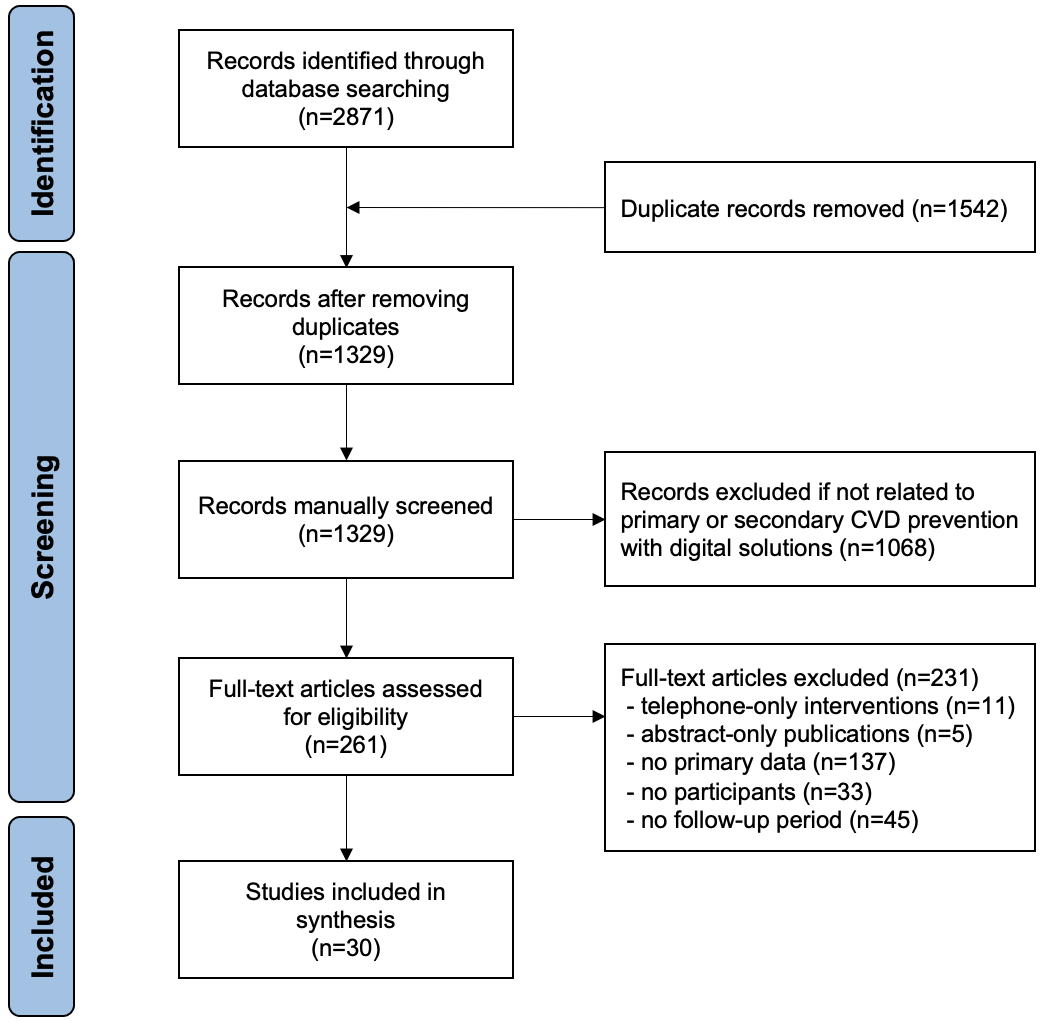
PRISMA (Preferred Reporting Items for Systematic Reviews and Meta-Analyses) flowchart. CVD: cardiovascular disease.

Following PRISMA guidelines, we conducted a comprehensive search on the Web of Science, Scopus, and PubMed, identifying a total of 2871 articles published in English. During the initial title and abstract screening, 1542 duplicate records were identified and removed, resulting in 1329 articles proceeding to the next stage. After further title and abstract review, 1068 articles were excluded due to their lack of relevance to telemedicine or digital solutions for primary or secondary CVD prevention, leaving 261 articles for full-text screening. Among these, 231 articles were subsequently excluded for reasons such as telephone-only interventions, abstract-only publications, no primary data, no participants, and no follow-up period. When articles met multiple exclusion criteria, they were classified under the first applicable category based on priority. Finally, 30 studies were included. A PRISMA flowchart of the screening process is provided in Figure 1.

### Characteristics of the Eligible Studies

**Figure 2:**
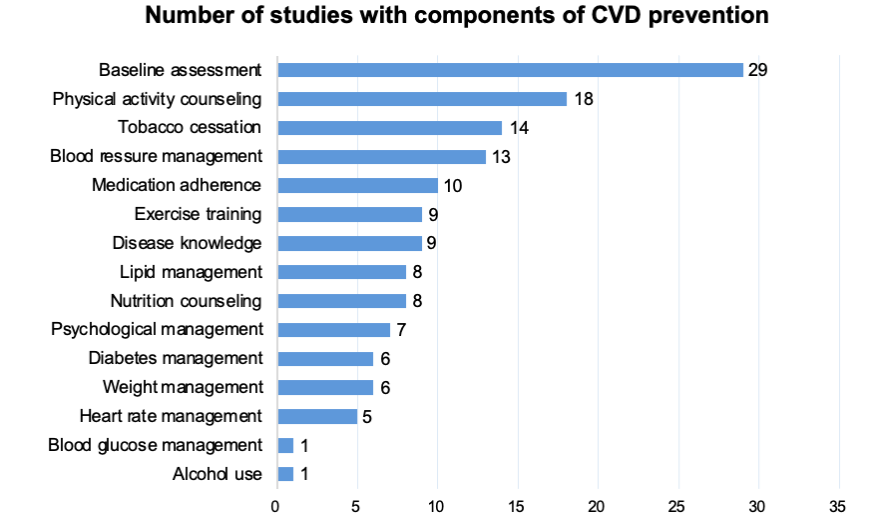
Number of eligible studies with components of CVD prevention in the systematic review. CVD: cardiovascular disease.

This review included 30 studies meeting the inclusion criteria detailed in Multimedia Appendix 3, with their characteristics detailed in Table 1. Of the 30 studies, 24 (80%) were randomized controlled trials, 6 (20%) were nonrandomized, and 23 (77%) focused on secondary prevention. The majority were conducted in the Americas (9/30, 30%). Of the 30 studies, 16 (53%) studies used smartphones and wearables, 12 (40%) studies used email or SMS communications, and 3 (10%, 3/30) studies used websites or web portals. Interventions are most often directed by research team staff (26/30, 87%), followed by health coaches (3/30, 10%), physicians (3/30, 10%), general health professionals (3/30, 10%), nurses (2/30, 7%), dietitians (1/30, 3%), pharmacists (1/30, 3%), and community health workers (1/30, 3%). The median sample size was 465.5 (IQR 162.75-751.5), with a median follow-up period of 6 (IQR 3.5-12) months and a median intervention duration of 6 (IQR 3-12) months. In total, 28 out of 30 (93%) studies were standalone digital CVD prevention interventions. A bar chart was used to compare the number of studies that included each component of CVD prevention (Figure 2). The most common components of CVD prevention were baseline assessment (29/30, 97%), physical activity counseling (18/30, 60%), tobacco cessation (14/30, 47%), blood pressure management (13/30, 43%), and medication adherence (10/30, 33%). Approximately one-third of the studies covered additional components of CVD prevention, such as disease knowledge (9/30, 30%) and exercise training (9/30, 30%). Less than one-third of the studies focused on nutrition counseling (8/30, 27%), lipid (8/30, 27%), psychological management (7/30, 23%), diabetes (6/30, 20%), weight (6/30, 20%), heart rate (5/30, 17%), blood glucose (1/30, 3%), and alcohol use (1/30, 3%).

**Table 1:** Summary of the eligible studies about digital solutions for CVD prevention in the systematic review.

Category and subcategory			Studies, n
**Research type**			
	Randomized controlled trial	24	
	Nonrandomized study	6	
**Prevention type**			
	Secondary	23	
	Primary	7	
**Location of study, by continent**			
	North and South America	9	
	Asia	8	
	Oceania	8	
	Europe	5	
**Publication year**			
	2015-2019	17	
	2020-2024	13	
**Technology use**			
	Smartphones and wearables	16	
	Email-SMS communications	12	
	Websites or web portals	3	
**Personnel**			
	Research team staffs	26	
	Health coaches	3	
	Physicians	3	
	General health professionals	3	
	Nurses	2	
	Dietitians	1	
	Pharmacists	1	
	Community health workers	1	

### Bias Reporting of the Eligible Studies

**Figure 3:**
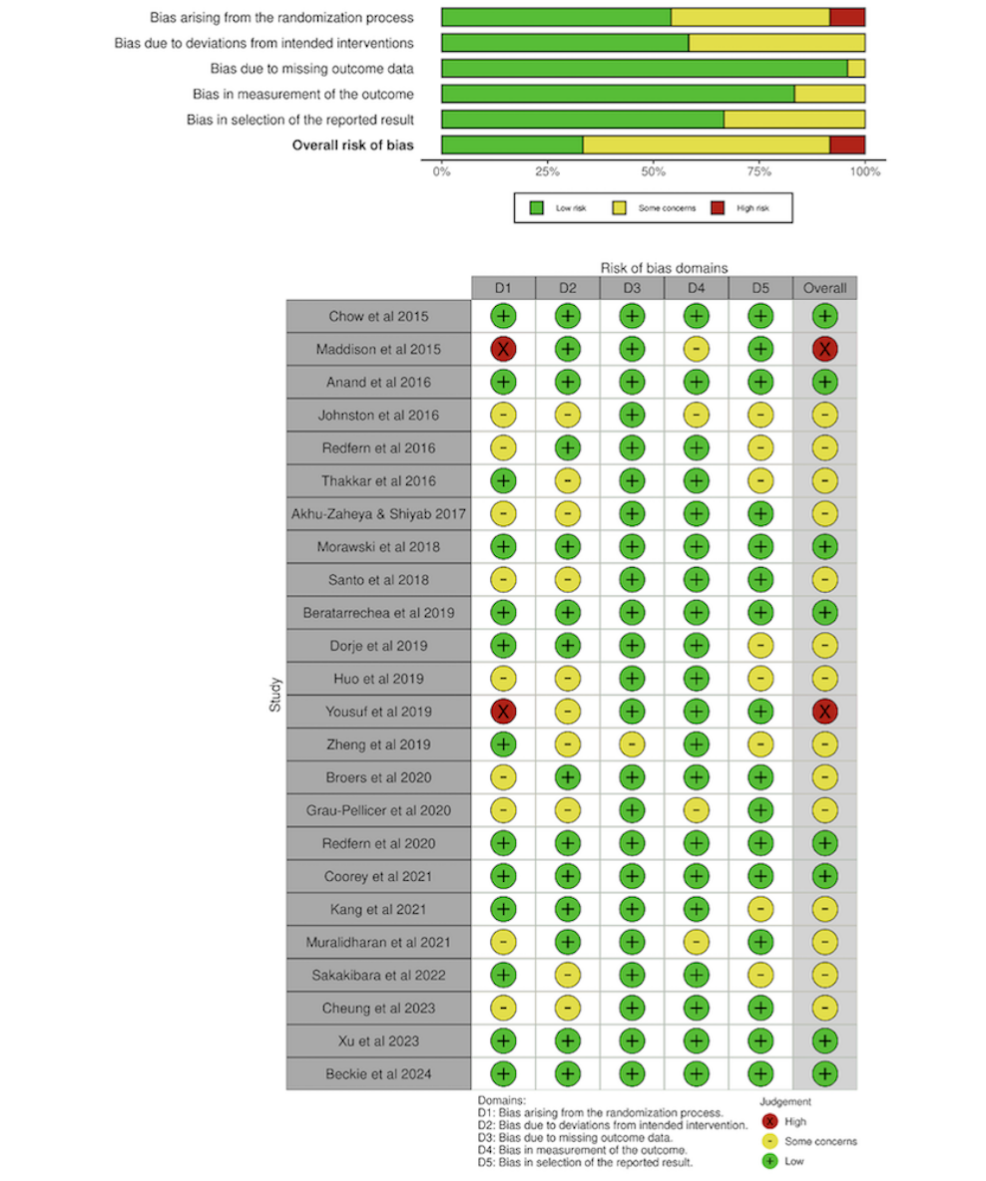
Risk of bias summary using RoB tool. RoB: risk of bias [17-23,25-27,29,30,32-36,38,39,40,42,43,45,46].

In the assessment of bias among the 24 randomized controlled trials using the RoB 2 tool, 2 [17,18] studies were found to have a high risk of bias, 14 [19-32] had some concerns for risk of bias, and 8 [33-40] were rated as having a low risk of bias. Although all studies applied randomization, 9 [19,20,22,23,25,27,28,30,32] did not clearly specify the implementation details of the randomization process, and 2 [17,18] reported that participants were not blinded to their allocation due to unavoidable research limitations. For the 6 nonrandomized studies, evaluated with the ROBINS-I tool, 1 [41] study was judged to have a serious risk of bias, 4 [42-45] were assessed as having moderate risk, and 1 [46] was rated as low risk. 4 [41-44] of these studies presented concerns due to insufficient control of confounding factors in before-and-after designs, and 1 [41] study exhibited issues with missing data. Figures 3 [17-40] and 4 [41-46] summarized the results of the risk of bias assessments for the eligible studies using the RoB 2 and ROBINS-I tools.

**Figure 4:**
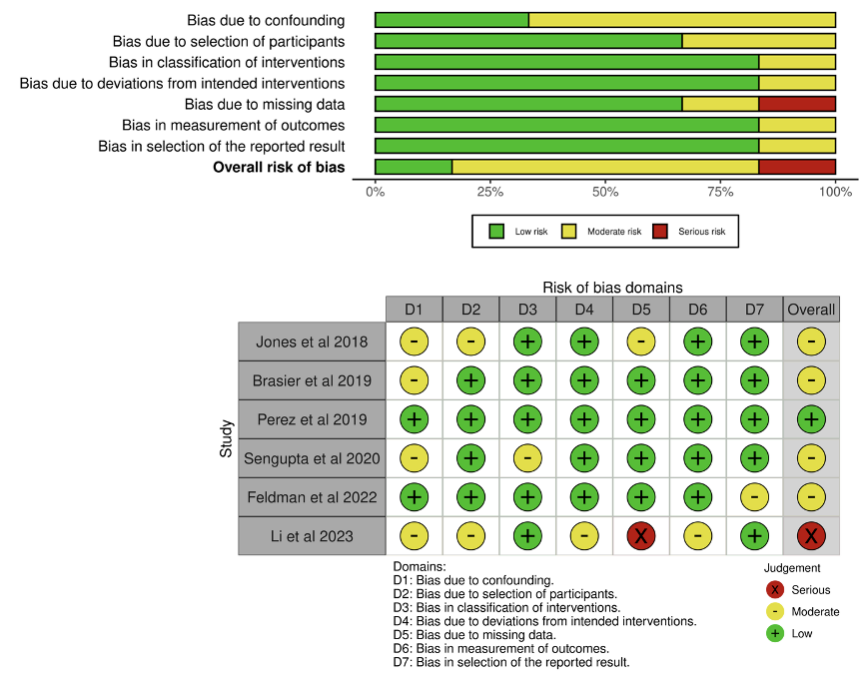
Risk of bias summary using the ROBINS-I tool [24,28,31,37,41,44].

### Technology Use in Digital Solutions

The analysis of 30 selected studies categorized the use of technology in digital health solutions for CVD prevention into 3 main categories: smartphones and wearables (53%, 16/30), email-SMS communications (40%, 12/30), and websites or web portals 10% (3/30). In addition, 3% (1/30) study incorporated both email-SMS communications and websites or web portals.

More than half of the selected studies integrated smartphones or mobile devices with wearable technology for monitoring and managing patients’ health conditions. Notable researchers including Johnston et al [19], Morawski et al [35], Beratarrechea et al [36], Brasier et al [43], Dorje et al [24], Perez et al [46], Grau-Pellicer et al [28], Sengupta et al, [44], Redfern et al [37], Broers et al [27], Kang et al [29], Muralidharan et al [30], and Li et al [41] focused on the efficacy of smartphone applications in preventing CVD. For instance, Johnston et al [19] evaluated an interactive patient support tool through a smartphone application designed to enhance treatment adherence and cardiovascular health among patients with myocardial infarction. According to Grau-Pellicer et al [28], they evaluated the effectiveness of a mHealth application on physical activity adherence and suggested that this technology provides a way to promote adherence to home exercise programs poststroke. Sengupta et al [44], Feldman et al [45], and Beckie et al [40] examined interventions that use smartphones and smartwatches to enhance CVD prevention. Xu et al [39] explored the smartphone-based gamification intervention for CVD prevention. The use of email-SMS communications was also considerable, indicating the popularity of interventions such as the TEXT ME program [20,21,23,26,33]. In addition, the use of email or SMS programs [18,25,34], and SMS intervention [22,32,42], illustrated the various approaches employed in the field. There was relatively limited research on the use of websites or portals. Coorey et al [38] introduced a purpose-built, versatile web-based application intervention. Sakakibara et al [31] developed an attention control memory training program delivered through the web. Maddison et al [17] took a multifaceted approach, integrating email-SMS communications and websites or portals into their interventions.

### Key Outcomes

Across the 30 studies, the most common key findings are summarized in Table 2. The most frequently evaluated outcome was blood pressure (14/30, 47%). Other commonly assessed outcomes included exercise capacity (12/30, 40%), weight (12/30, 40%), lipid profile (11/30, 37%), nicotine dependence (9/30, 30%), medication use (8/30, 27%), quality of life (7/30, 23%), dietary habits (5/30, 17%), intervention adherence (4/30, 13%), waist (4/30, 13%), and blood glucose (2/30, 7%). We have distinguished between findings related to enhancing preventive behaviors and those related to monitoring health indicators for a more nuanced understanding of the results. For instance, blood pressure was categorized as a health indicator, whereas exercise capacity was categorized as a preventive behavior for CVD prevention. After the analysis of the key findings, it was found that individuals using digital solutions generally performed better when receiving interventions compared with individuals using traditional CVD prevention measures. For instance, due to reminders and personalized feedback, digital interventions promoted more consistent medication use [19,22,32,35,36,41]. Furthermore, as a result of the enhanced engagement and support provided by these technologies, the quality of life indicators of participants using digital solutions improved significantly [17,18,27,28,31]. Dietary habits had also improved substantially, with digital platforms often providing tailored nutrition advice and monitoring that was more effective than traditional measures [18,22-24,40]. Intervention adherence was notably higher, which could be attributed to the real-time feedback provided by digital tools [19,26,41,44]. Waist circumference also showed a slight reduction among those using digital interventions [30,40,42].

**Table 2:** Summary of key findings by thematic outcomes.

Key findings	Studies, n	Summary
Blood pressure (health indicator)	14	8 of the 14 studies reported that the digital solutions improved blood pressure management and were no worse than the control group [18,20,24,33,35,37,40,42]. In contrast, 6 studies concluded that it did not have a statistically significant influence on blood pressure management [25,26,29,30,32,34].
Exercise capacity (preventive behavior)	12	7 studies examined exercise capacity as outcomes showing that the performance of the intervention group was comparable to that of the control group [20,21,24,28,33,37,39]. Notably, in 5 of these studies, no significant difference in exercise capacity was observed [17-19,25,26].
Weight (preventive behavior)	12	7 of the 12 studies, digital solutions were effective in addressing weight management [18,20,23,33,39,42,44]. However, 5 studies found no significant change or a slight decrease in body weight or BMI after the digital intervention [19,24-26,37].
Lipid profile (health indicator)	11	4 of the 11 studies reported elevated high-density lipoprotein levels in the intervention group [18,23,24,37]. 3 other studies showed reductions in low-density lipoprotein and total cholesterol levels [20,33,42]. No significant changes were observed in the remaining 4 studies [25,26,29,32].
Nicotine dependence (health indicator)	9	Of the 9 studies included, 4 showed a positive trend towards improvement in smoking habits, as evidenced by a reduction in nicotine dependence scores [20,29,33,36], while the remaining 5 did not change significantly [19,22,24,26,37].
Medication use (preventive behavior)	8	6 studies found that medication use was beneficial and had a positive effect [19,22,32,35,36,41]. However, 2 studies reported slight or insignificant changes due to medication [37,45].
Quality of life (preventive behavior)	7	5 studies documented an improvement in quality of life for participants in the intervention group [17,18,27,28,31]. Conversely, 2 studies failed to observe significant differences in quality of life [19,24].
Dietary habits (preventive behavior)	5	Of the 5 studies reviewed, all reported improvements in dietary habits [18,22-24,40].
Intervention adherence (preventive behavior)	4	4 studies provided evidence to support the idea that digital solutions can improve individuals' adherence to interventions [19,26,41,44].
Waist (health indicator)	4	3 studies showed a slight reduction in waist circumference after the digital intervention [30,40,42]. However, there was also 1 study in which no significant effects were observed [34].
Blood glucose (health indicator)	2	1 study showed the effectiveness of interventions in managing blood glucose levels [25]. In contrast, 1 study found no significant benefit in this regard [18].

Notably, the digital solution showed efficacy comparable to the traditional control group across outcomes, including nicotine dependence, blood pressure, blood glucose levels, exercise capacity, and weight management. For example, digital interventions had the same impact on nicotine dependence as traditional interventions, with nearly half of the studies showing a positive trend toward improving smoking habits [20,29,33,36], and the remaining half showing no significant change [19,22,24,26,37]. Similarly, blood pressure, blood glucose levels, exercise capacity, and weight management results were comparable to those of the traditional control group. However, the effectiveness of digital solutions varies when it comes to lipid profiles. Of the 11 studies reviewed, 4 reported elevated high-density lipoprotein levels in the intervention group [18,23,24,37]. 3 other studies showed reductions in low-density lipoprotein and cholesterol levels [20,33,42]. No significant changes were observed in the remaining 4 studies [25,26,29,32].

Study outcomes varied across follow-up times. Dietary habits and intervention adherence yielded positive results at various intervals, including 3, 6, and 12 months [18,22-24,40], as well as 3, 5, 6, and 8 months [19,26,41,44]. Waist circumference decreased at 3 months in studies by Beckie et al [40], Jones et al [42], and Muralidharan et al [30], but Anand et al [34] found no significant effects at 12 months. Quality of life was assessed at 6-month intervals [17,18,27,31] and at 2 months [28], with Johnston et al [19] and Dorje et al [24] finding no significant differences in the digital intervention versus usual care groups at 6 and 12 months. Medication uses improved health outcomes over 3, 5, 6, and 12 months [19,22,32,35,36,41], although Feldman et al [45] and Redfern et al [37] observed minimal changes over longer periods (12 and 43 months). Studies on nicotine dependence showed varied results, with some studies indicating positive trends over longer follow-ups [20,29,33,36], while others reported different outcomes [19,22,24,26,37]. Positive blood pressure outcomes were reported within 3 months by Beckie et al [40], Jones et al [42], and Morawski et al [35], with improvements observed at 6 months by Chow et al [33] and Yousuf et al [18]. Dorje et al [24] and Redfern et al [20,37] noted significant benefits at 12 months, whereas Anand et al [34], Kang et al [29], and Cheung et al [32] respectively found no significant impact at 12 and 6 months. Muralidharan et al [30], Huo et al [25], and Zheng et al [26] did not observe notable improvements over 3, 6, and 8 months. Few studies exist on blood glucose levels, with Huo et al [25] showing effectiveness at 6 months, but Yousuf et al [18] found no significant benefits during the same period. For exercise capacity, positive findings were reported at 6 and 12 months by Chow et al [33], Thakkar et al [21], Dorje et al [24], and Redfern et al [20,37], with shorter follow-ups also supporting these findings. However, no significant differences were found between digital intervention and usual care groups at 6 or 8 months [17-19,25,26]. For weight management, significant reductions were noted at 6 and 12 months by Chow et al [33] and Redfern et al [20], while Jones et al [42] and Sengupta et al [44] reported favorable outcomes at 3 months. However, minimal to no improvements were found by Dorje et al [24] and Huo et al [25] at 6 months. Johnston et al [19] and Redfern et al [37] also showed no significant changes at 6 and 12 months, respectively. Studies with 3 to 6-month follow-ups predominantly showed no significant changes in lipid profiles, with minimal impacts observed within 6 months [23,32,42]. Conversely, longer follow-ups tended to show more pronounced effects, such as significant increases in high-density lipoprotein reported by Dorje et al [24] and Redfern et al [37] and reductions in low-density lipoprotein and total cholesterol observed by Redfern et al [20] and Chow et al [33]. However, Huo et al [25] and Kang et al [29] found no significant lipid changes in long-term studies.

The review examined the comprehensive standalone interventions and found that the interventions in 7 studies identified as standalone included 5 components or more for CVD prevention, excluding baseline assessments [18,23,24,26,29,30,37]. Outcomes associated with most of the key outcomes associated with the prevention of CVD components were heterogeneous throughout the intervention. Among these 7 studies, Redfern et al [37] documented outcomes regarding medication use, showing no significant effects. Regarding blood glucose, Yousuf et al [18] did not find a significant benefit. With regard to waist circumference, Muralidharan et al [30] demonstrated a reduction in waist circumference after the digital intervention. Santo et al [23], Dorje et al [24], and Yousuf et al [18] observed improvements in mood within the intervention group, while Redfern et al [37] found similar results. Nevertheless, Kang et al [29] and Zheng et al [26] discovered that the digital solutions did not significantly impact lipid profiles. Regarding weight management, Santo et al [23], Yousuf et al [18], and Redfern et al [37] observed that digital solutions were effective. In contrast, Dorje et al [24] and Zheng et al [26] reported that there was no significant change, or even a slight decrease, in body weight or BMI after the digital intervention. Santo et al [23], Dorje et al [24], and Yousuf et al [18] all recorded improvements in dietary habits. Dorje et al [24], Yousuf et al [18], Redfern et al [37], and Muralidharan et al [30] reported that the digital solution significantly enhanced blood pressure management and was no worse than the control group. However, Kang et al [29] found that digital solutions did not significantly affect blood pressure management. In terms of exercise capacity, Dorje et al [24] and Redfern et al [37] showed that the intervention group was similar to the control group. Yousuf et al [18] and Zheng et al [26] discovered that there was no difference in exercise capacity between the digital intervention group and the control group. For nicotine dependence, Kang et al [29] showed a positive trend toward improving smoking habits, while Dorje et al [24], Zheng et al [26], and Redfern et al [37] showed no significant change. In terms of quality of life, Yousuf et al [18] reported enhanced quality of life, while Dorje et al [24] reported no significant difference in the quality of life of intervention groups. Overall, interventions taken and reported outcomes varied.

## Discussion

### The Role of Digital Solutions for CVD Prevention

The study highlights the potential of digital technologies to enhance health care delivery and expand CVD prevention by enhancing preventive behaviors and monitoring health indicators. These digital solutions can significantly improve individual outcomes and facilitate the wider implementation of preventive approaches. Unlike previous systematic reviews, which often focused narrowly on specific aspects of digital health, our study provides a comprehensive assessment of digital solutions, including primary and secondary CVD prevention. Our review demonstrates the varied effectiveness of these digital solutions. However, variations in potential risks of bias, as assessed, should be considered when interpreting the findings. Based on existing studies, our review supports remote CVD prevention through internet-based platforms and digital devices, which offer innovative ways to monitor, educate, and engage individuals. Our findings show that using digital solutions to prevent CVD is both feasible and effective, and the results are comparable to traditional approaches. Whether as a complement or substitute for traditional approaches, digital solutions show great potential in improving individual health outcomes [19,21-28,30,33,35,36,39-43]. Notably, the integration of digital technologies into CVD prevention can make more efficient use of health care resources, reduce the burden on health care systems, and provide cost-effective solutions for individuals [17,18].

The results of our review are consistent with previous studies, including Gray et al [47], which demonstrated the effectiveness of remote consultation, smartphone applications, wearables, remote monitoring, and predictive analytics in influencing individual behavior. These digital solutions can greatly contribute to the primary and secondary prevention of CVD and play a role in preventing and managing disease. In addition, Moshawrab et al [48] showed that wearable devices were highly accurate in detecting, predicting, and even treating CVD. Their study highlighted the potential of digital health solutions to improve individual outcomes and optimize the use of health care resources, further supporting their integration into standard CVD prevention strategies. Our review and the included studies highlight the role of effective communication strategies in the success of digital health solutions for CVD prevention. Effective communication influences user engagement, adherence, and the overall impact of these interventions. Specific strategies, such as using interactive features to boost engagement, providing reminders to support adherence, offering support through virtual coaching, tailoring experiences to individual needs, and implementing feedback mechanisms, can significantly motivate individuals to participate and commit to preventive behaviors. By fostering a supportive environment, effective communication strengthens relationships between health care providers and patients, empowering individuals to take an active role in managing their health and promoting health outcomes. However, the effectiveness of communication strategies in digital health solutions is hindered by barriers related to digital literacy and access. Many individuals, particularly those at higher risk of CVD, may lack the skills required to use digital tools effectively. Without sufficient digital literacy, the potential benefits of remote consultations, smartphone applications, wearables, and remote monitoring systems may remain inaccessible for some individuals. The digital divide, marked by disparities in digital skills and access, poses a significant obstacle to the equitable and effective implementation of digital health solutions [49]. To address these barriers, targeted strategies to improve digital literacy are essential. Initiatives may include specialized training programs designed to help users develop the skills necessary to engage effectively with digital health tools, with a focus on older adults and underserved populations who may face greater challenges. Furthermore, designing user-friendly interfaces and simplifying onboarding processes can facilitate initial engagement and reduce usability obstacles. Health care providers also play a role in offering technical support, instructions, and tutorials that guide users in understanding and integrating digital tools into their daily routines. Enhancing digital literacy and access is important to enabling effective communication, thereby amplifying the impact of digital health solutions for CVD prevention. By improving digital skills and accessibility, individuals can more effectively set baselines, customize treatment plans, monitor progress, and receive tailored support, which strengthens their capacity to engage with preventive behaviors and optimize health outcomes.

### Limitations and Future Research Recommendations

Some limitations appeared in our review. First, although this review aimed to address the effects and factors surrounding specific phenomena, we did not use meta-analysis methods to summarize empirical evidence due to the heterogeneity of the studies. The reviewed articles exhibited significant variation in study designs, main outcomes, technologies, and control groups. For instance, some studies focused on a single digital health intervention, such as a mobile health application, while others looked at a combination or multiple uses of various digital technologies. In addition, when considering control groups, some studies used standard care in people with traditional CVD prevention, while others did not include control groups. Second, this review used RoB 2 and ROBINS-I tools to assess the risk of bias in the included studies. While both tools were widely recognized for evaluating bias, they might be influenced by subjective judgment, particularly in complex cases where interpretations of criteria can vary among evaluators. Third, our review was limited in scope and time frame. While we included studies published between 2000 and 2024, the earliest studies meeting our inclusion criteria were from 2015, likely because digital solutions for disease prevention represent a relatively new field that has emerged with recent technological advances. Furthermore, our search was limited to 3 databases and did not include reference list searches and gray literature. As this is a rapidly evolving field, studies published after the search date may have been missed. Fourth, our review only highlighted primary and secondary prevention of CVD. However, tertiary prevention, which involved advanced medical procedures and interventions aimed at managing and mitigating the long-term effects of diagnosed CVD, also played a role in the integrated management of CVD. Therefore, we suggested several strategies for future research. First, it is crucial that future studies standardize the objectives of interventions to ensure consistency in study design, outcome measures, and control groups. Specifically, researchers should clearly define whether their digital solutions are intended as comprehensive standalone programs or as complementary tools to traditional prevention approaches. Second, future research should also aim to standardize and provide detailed descriptions of the specific CVD prevention components targeted by the intervention. This includes specifying whether the intervention’s goal is to enhance preventive behaviors, monitor key health indicators (eg, blood pressure and cholesterol), or address both aspects simultaneously. Third, it is essential to incorporate robust measures of user engagement and behavior modification in evaluating digital solutions. Researchers should use validated metrics such as compliance rates, user satisfaction surveys, and long-term health outcomes to assess the impact of digital interventions on user behavior and sustained engagement. Finally, exploring the cost-effectiveness of digital solutions for CVD prevention, including tertiary prevention, compared to traditional approaches is crucial. Future studies should assess not only direct costs but also factors such as scalability, maintenance, and initial setup costs.

We have identified several key aspects for future research in the field of digital interventions for CVD prevention. First, while digital solutions effectively address factors such as eating habits, interventions targeting other risk factors, including blood glucose control, are still lacking. Moreover, further evaluation is needed to assess the ongoing efficacy of these digital solutions, so studies using a larger population and implementing suitable control groups are needed [8,50]. Second, future research should focus on integrating multifaceted interventions to address a wide range of CVD risk factors, including stress management. It is crucial to study the synergies of combining digital tools such as mobile apps, wearables, and telemedicine. Understanding the psychological and behavioral mechanisms behind user engagement, such as motivation, digital literacy, and personalization, is also critical to improving adherence and sustained behavior change [51]. Third, it is crucial to evaluate the incidence of adverse events among individuals undergoing these interventions compared to traditional methods [14]. Rigorous monitoring and reporting of adverse events will provide insight into the potential risks associated with these technologies, helping to refine protocols and ensure individual safety. Finally, exploring the potential of artificial intelligence (AI) to improve communication effectiveness by personalizing and tailoring interventions in real time could significantly improve user experience [52,53]. As an emerging field, AI has shown great potential in fields such as health care and education. In the field of CVD prevention, its ability to analyze large datasets, predict individual outcomes, and tailor interventions to individual health conditions holds great potential for strengthening prevention measures and optimizing patient care pathways [52-54]. As research in this field continues to advance, the integration of AI into clinical practice could lead to more effective, personalized, and accessible solutions to combat CVD worldwide.

### Conclusions

Our study highlights the potential of digital technologies to alleviate challenges associated with traditional CVD prevention approaches by enhancing preventive behaviors and monitoring health indicators. The widespread implementation of digital solutions in CVD prevention is expected to have a significant impact, increasing accessibility, affordability, and cost-effectiveness, and improving individual outcomes beyond what can be achieved with traditional approaches. However, interventions evaluated focused primarily on medication use, quality of life, dietary habits, intervention adherence, and waist circumference, with limited attention to other components of CVD prevention. In addition, our study primarily assessed the technical aspects and comprehensiveness of digital procedures in the prevention of CVD. However, the complexities associated with the certification of CVD prevention programs are beyond the scope of this review. Future studies should aim to explore more comprehensive CVD prevention interventions to assess their long-term and sustained impact.

## Appendix

Multimedia Appendix 1 PRISMA (Preferred Reporting Items for Systematic Reviews and Meta-Analyses) 2020 checklist.

Multimedia Appendix 2 Search Terms.

Multimedia Appendix 3 Characteristics of the eligible studies about digital solutions for CVD prevention in the systematic review.

## Abbreviations

AI: artificial intelligence
CVD: cardiovascular disease
mHealth: mobile health
PICO: Population, Intervention, Comparison, Outcome
PRISMA: Preferred Reporting Items for Systematic Reviews and Meta-Analyses
RoB: Risk of Bias

## Data Availability

All data generated or analyzed during this study are included in this published article and its supplementary information files.
