# Supplementary material for: Digital Health Solutions for Cardiovascular Disease Prevention: Systematic Review
Source: J Med Internet Res. 2025 Jan 23;27:e64981. doi: 10.2196/64981 (PMC11803337; doi:10.2196/64981)
Supplement: Multimedia Appendix 2 [file jmir_v27i1e64981_app2.docx]

**Search Terms**

**Web of Science**

TS=("Digital Health" OR "eHealth" OR "mHealth" OR "Mobile Health" OR "Text Message" OR "Text" OR "Short Message Service" OR "SMS" OR "Mobile Application" OR "Telemedicine")

AND

TS=("Prevention" OR "Prevent")

AND

TS=("Cardiovascular Disease" OR "CVD" OR "Heart Disease" OR "Cardiovascular Health" OR "Cardiovascular Risk Factors" OR "Heart Attack" OR "Stroke" OR "Cardiac Arrest" OR "Heart Condition" OR "Heart Health")

**Scopus**

TITLE-ABS-KEY("Digital Health" OR "eHealth" OR "mHealth" OR "Mobile Health" OR "Text Message" OR "Text" OR "Short Message Service" OR "SMS" OR "Mobile Application" OR "Telemedicine")

AND

TITLE-ABS-KEY("Prevention" OR "Prevent")

AND

TITLE-ABS-KEY("Cardiovascular Disease" OR "CVD" OR "Heart Disease" OR "Cardiovascular Health" OR "Cardiovascular Risk Factors" OR "Heart Attack" OR "Stroke" OR "Cardiac Arrest" OR "Heart Condition" OR "Heart Health")

**PubMed**

("Digital Health"[Title/Abstract] OR "eHealth"[Title/Abstract] OR "mHealth"[Title/Abstract] OR "Mobile Health"[Title/Abstract] OR "Text Message"[Title/Abstract] OR "Text"[Title/Abstract] OR "Short Message Service"[Title/Abstract] OR "SMS"[Title/Abstract] OR "Mobile Application"[Title/Abstract] OR "Telemedicine"[Title/Abstract])

AND

("Prevention"[Title/Abstract] OR "Prevent"[Title/Abstract])

AND

("Cardiovascular Disease"[Title/Abstract] OR "CVD"[Title/Abstract] OR "Heart Disease"[Title/Abstract] OR "Cardiovascular Health"[Title/Abstract] OR "Cardiovascular Risk Factors"[Title/Abstract] OR "Heart Attack"[Title/Abstract] OR "Stroke"[Title/Abstract] OR "Cardiac Arrest"[Title/Abstract] OR "Heart Condition"[Title/Abstract] OR "Heart Health"[Title/Abstract])
