# Supplementary material for: Digital Health Solutions for Cardiovascular Disease Prevention: Systematic Review
Source: J Med Internet Res. 2025 Jan 23;27:e64981. doi: 10.2196/64981 (PMC11803337; doi:10.2196/64981)
Supplement: Multimedia Appendix 3 [file jmir_v27i1e64981_app3.docx]

**Table**. Characteristics of the eligible studies about digital solutions for CVD prevention in the systematic review.

| Reference | Prevention Type | Location | Research Design and Participation | Intervention Measures | Components of CVD prevention | Personnel and Delivery Setting | Outcomes | Follow-up and Intervention Duration |
| --- | --- | --- | --- | --- | --- | --- | --- | --- |
| Chow et al., 2015 [1] | Secondary | Australia | RCT among 710 patients with documented coronary heart disease (CHD) | 6-month intervention of tobacco, exercise and diet messages (TEXT ME) program | - Baseline assessment - Tobacco cessation - Physical activity counseling - Nutrition counseling - Disease knowledge | - Standalone intervention - Researchers | - Lipid profile - Blood pressure - Weight - Exercise capacity - Nicotine dependence | - 6 months - 6 months |
| Maddison et al., 2015 [2] | Secondary | New Zealand | RCT among 171 patients with CHD | 24-week heart exercise and remote technologies intervention about personalized, automated package of text messages, and secure website with video messages | - Baseline assessment - Exercise training - Psychological management | - Standalone intervention - Researchers, staff | - Oxygen uptake - Exercise capacity - Self-efficacy - Quality of life - Economic evaluation | - 6 months - 6 months |
| Anand et al., 2016 [3] | Primary | Canada | RCT with 343 people who were free of cardiovascular disease (CVD) | 12-month emails or text messages program | - Baseline assessment - Physical activity counseling - Nutrition counseling | - Standalone intervention - Researchers | - Myocardial infarction - Blood pressure - Waist | - 12 months - 12 months |
| Johnston et al., 2016 [4] | Secondary | Sweden | RCT among 174 myocardial infarction patients | 6-month interactive patient support application on phone | - Baseline assessment - Weight management - Exercise training - Tobacco cessation - Blood pressure management - Blood glucose management | - In-person session - Standalone intervention - Nurses, physicians | - Weight - Exercise capacity - Nicotine dependence - Medication use - Intervention adherence - Quality of life - Satisfaction | - 6 months - 6 months |
| Redfern et al., 2016 [5] | Secondary | Australia | RCT among 1301 patients with CHD | 6-month text messages intervention of TEXT ME program | - Baseline assessment - Tobacco cessation - Physical activity counseling - Nutrition counseling - Disease knowledge | - In-person session - Standalone intervention - Group session - Researchers | - Lipid profile - Blood pressure - Weight - Exercise capacity - Nicotine dependence | - 12 months - 6 months |
| Thakkar et al., 2016 [6] | Secondary | Australia | RCT among 710 patients with CHD | 6-month text messages intervention of TEXT ME program | - Baseline assessment - Tobacco cessation - Physical activity counseling - Nutrition counseling - Disease knowledge | - Standalone intervention - Researchers | - Exercise capacity | - 6 months - 6 months |
| Akhu-Zaheya & Shiyab, 2017 [7] | Secondary | Jordan | RCT among 160 patients with CVD | 3-month short message service (SMS) intervention | - Baseline assessment - Tobacco cessation - Medication adherence - Nutrition counseling | - Standalone intervention - Researchers | - Medication use - Dietary habits - Nicotine dependence | - 6 months - 3 months |
| Jones et al., 2018 [8] | Primary | United States | Non-randomized study among 40 people at risk for CVD | 12-week SMS and longer text messages intervention | - Baseline assessment - Blood pressure management - Weight management - Lipid management - Exercise training - Medication adherence - Nutrition counseling | - In-person session - Group session - Researchers | - Weight - Waist - Lipid profile - Blood pressure | - 3 months - 3 months |
| Morawski et al., 2018 [9] | Primary | United States | RCT among 411 hypertensive patients | 12-week smartphone application intervention | - Baseline assessment - Blood pressure management - Medication adherence | - Standalone intervention - Researchers | - Blood pressure - Medication use | - 3 months - 3 months |
| Santo et al., 2018 [10] | Secondary | Australia | RCT among 710 patients with CHD | 6-month intervention of TEXT ME | - Baseline assessment - Nutrition counseling - Blood pressure management - Lipid management - Weight management - Tobacco cessation - Physical activity counseling | - Standalone intervention - Researchers | - Lipid profile - Weight - Dietary habits | - 6 months - 6 months |
| Beratarrec-hea et al., 2019 [11] | Primary | Argentina | RCT among 755 people at risk for CVD | 6-month mHealth application intervention for calculating CVD risk | - Baseline assessment - Blood pressure management - Disease knowledge | - Standalone intervention - Study administrative staff, healthcare professional-s, community health workers | - Medication use - Health outcomes - Nicotine dependence | - 12 months - 6 months |
| Brasier et al., 2019 [12] | Secondary | Switzerland and Germany | Non-randomized study among 672 patients with presumed atrial fibrillation (AF) | 12-month smartphones intervention with installed a study version of the commercially available Heartbeats application | - Baseline assessment - Heart rate management | - Standalone intervention - Researchers, cardiology physicians | - Detection of AF | - 12 months - 12 months |
| Dorje et al., 2019 [13] | Secondary | China | RCT among 321 patients with CHD | 4-month intervention via WeChat | - Baseline assessment - Tobacco cessation - Exercise training - Physical activity counseling - Blood pressure management - Heart rate management - Disease knowledge - Medication adherence - Psychological management | - Standalone intervention - Social media platform WeChat coaches | - Health outcomes - Lipid profile - Blood pressure - Exercise capacity - Nicotine dependence - Dietary habits - Weight - Quality of life | - 12 months - 6 months |
| Huo et al., 2019 [14] | Secondary | China | RCT among 502 patients with both CHD and diabetes mellitus | 6-month text messages intervention | - Baseline assessment - Blood pressure management - Lipid management - Medication adherence - Physical activity counseling | - Standalone intervention - Researchers | - Blood pressure - Blood glucose - Lipid profile - Weight - Exercise capacity | - 6 months - 6 months |
| Perez et al., 2019 [15] | Primary | United States | Non-randomized study among 419297 participants without AF | 3-month smartphone application intervention | - Baseline assessment - Heart rate management | - Standalone intervention - Researchers | - Pulse | - 3 months - 3 months |
| Yousuf et al., 2019 [16] | Primary | Netherlands and United Kingdom | RCT among 402 people at risk for CVD | 18-month email, e-Coaching intervention | - Baseline assessment - Blood pressure management - Lipid management - Weight management - Physical activity counseling - Diabetes management | - In-person session - Standalone intervention - Group session - Researchers | - Weight - Blood pressure - Blood glucose - Lipid profile - Quality of life - Exercise capacity - Dietary habits - Economic evaluation | - 6 months - 18 months |
| Zheng et al., 2019 [17] | Secondary | China | RTC among 822 patients with CHD | 6-month text messages intervention of TEXT ME | - Baseline assessment - Disease knowledge - Medication adherence - Blood pressure management - Tobacco cessation - Exercise training - Physical activity counseling - Diabetes management | - In-person session - Standalone intervention - Group session - Researchers | - Blood pressure - Weight - Nicotine dependence - Lipid profile - Exercise capacity | - 8 months - 6 months |
| Broers et al., 2020 [18] | Secondary | Spain and the Nether-lands | RCT among 150 patients with CHD | 3-month behavioral intervention eHealth program | - Baseline assessment - Exercise training - Physical activity counseling - Diabetes management | - Standalone intervention - Researchers | - Life style - Quality of life - Satisfaction - Intervention adherence | - 6 months - 3 months |
| Grau-Pellicer et al., 2020 [19] | Secondary | Spain | RCT among 41 patients with stroke | 8-week intervention about digital platform based on mHealth applications | - Baseline assessment - Exercise training - Physical activity counseling | - Standalone intervention - Group session - Researchers | - Community ambulation - Sedentary behavior - Exercise capacity - Quality of life - Satisfaction | - 2 months - 2 months |
| Redfern et al., 2020 [20] | Secondary | Australia | RCT among 934 patients with or at risk of CVD | 12-month Consumer Navigation of Electronic Cardiovascular Tools intervention | - Baseline assessment - Medication adherence - Disease knowledge - Exercise training - Physical activity counseling - Diabetes management - Tobacco cessation - Psychological management | - In-person session - Standalone intervention - Researchers, clinical staff | - Medication use - Blood pressure - Lipid profile - Nicotine dependence - Weight - Exercise capacity - eHealth literacy - All-cause mortality | - 12 months - 12 months |
| Sengupta et al., 2020 [21] | Secondary | United States | Non-randomized study among 10 women | 12-week intervention via smartphone and smartwatch on which the HerBeat application | - Baseline assessment - Exercise training - Heart rate management - Diabetes management - Psychological management | - Standalone intervention - Researchers, trained professional-s, health coaches | - Intervention adherence - Weight - Anxiety or depression | - 3 months - 3 months |
| Coorey et al., 2021 [22] | Secondary | Australia | RCT among 934 patients with CVD | 12-month purpose-built, multi-feature web application intervention | - Baseline assessment - Nutrition counseling - Psychological management - Tobacco cessation - Physical activity counseling - Medication adherence - Disease knowledge | - In-person session - Standalone intervention - Nurses, dietitians, pharmacists | - Life style - eHealth literacy | - 12 months - 12 months |
| Kang et al., 2021 [23] | Secondary | Korea | RCT among 666 patients with clinical atherosclerotic CVD | 6-month smartphone application intervention | - Baseline assessment - Blood pressure management - Physical activity counseling - Weight management - Medication adherence - Lipid management - Tobacco cessation | - Standalone intervention - Researchers, physicians | - CVD Risk - Blood pressure - Lipid profile - Nicotine dependence - Diabetes status | - 12 months - 6 months |
| Muralidhar-an et al., 2021 [24] | Primary | India | RCT among 741 people with prediabetes or obesity | 12-week intervention about mDiab application in smartphones and weekly health coach calls | - Baseline assessment - Blood pressure management - Physical activity counseling - Weight management - Lipid management - Tobacco cessation | - Standalone intervention - Group session - Researchers, coaches | - Waist - Blood pressure | - 3 months - 3 months |
| Feldman et al., 2022 [25] | Secondary | United States | Non-randomized study  among 1802 patients with stroke | 43-month medical history, Apple Watch wear patterns, and AF risk intervention | - Blood pressure management - Weight management - Heart rate management - Tobacco cessation - Alcohol use | - Standalone intervention - Group session - Researchers | - Medication use | - 43 months - 43 months |
| Sakakibara et al., 2022 [26] | Secondary | Canada | RCT among 126 patients within one-year post-stroke | 12-month intervention about Stroke Coach and attention control Memory Training | - Baseline assessment - Physical activity counseling - Medication adherence - Blood pressure management - Lipid management | - Standalone intervention - Group session - Researchers, health professionals | - Life style - Quality of life - Cognitive status - Anxiety or depression | - 6 months - 6 months |
| Cheung et al., 2023 [27] | Secondary | Australia | RCT among 902 patients with type 2 diabetes or CHD | 6-month SMS text messages intervention | - Baseline assessment - Lipid management - Tobacco cessation - Physical activity counseling - Diabetes management | - In-person session - Researchers | - Blood pressure - Lipid profile - Medication use - Engagement | - 6 months - 6 months |
| Li et al., 2023 [28] | Secondary | China | Non-randomized study  among 243 patients with stroke | 3-month smartphone mobile application intervention | - Baseline assessment - Disease knowledge - Medication adherence | - In-person session - Standalone intervention - Researchers | - Intervention adherence - Medication use | - 5 months - 3 months |
| Xu et al., 2023 [29] | Secondary | China | RCT among 108 patients with CHD | 12-week smartphone-based gamification intervention | - Baseline assessment - Physical activity counseling - Psychological management | - Standalone intervention - Researchers | - Step count - Weight - Exercise capacity - Competence - Engagement | - 3 months - 3 months |
| Beckie et al., 2024 [30] | Secondary | United States | RCT among 47 women with CHD | 3-month mHealth intervention with a smartphone, smartwatch, and health coach | - Baseline assessment - Physical activity counseling | - In-person session - Standalone intervention - Researchers | - Dietary habits - Anxiety or depression - Self-efficacy - Blood pressure - Health outcomes - Waist | - 3 months - 3 months |

AF: atrial fibrillation

CHD: coronary heart disease

CVD: cardiovascular disease

RCT: randomized controlled trial

SMS: short message service

TEXT ME: tobacco, exercise and diet messages

Reference

1. Chow CK, Redfern J, Hillis GS, Thakkar J, Santo K, Hackett ML, Jan S, Graves N, de Keizer L, Barry T, Bompoint S, Stepien S, Whittaker R, Rodgers A, Thiagalingam A. Effect of Lifestyle-Focused Text Messaging on Risk Factor Modification in Patients With Coronary Heart Disease: A Randomized Clinical Trial. JAMA 2015 Sep 22;314(12):1255–1263. doi: 10.1001/jama.2015.10945

2. Maddison R, Pfaeffli L, Whittaker R, Stewart R, Kerr A, Jiang Y, Kira G, Leung W, Dalleck L, Carter K, Rawstorn J. A mobile phone intervention increases physical activity in people with cardiovascular disease: Results from the HEART randomized controlled trial. Eur J Prev Cardiol SAGE Publications Ltd STM; 2015 Jun 1;22(6):701–709. doi: 10.1177/2047487314535076

3. Anand SS, Samaan Z, Middleton C, Irvine J, Desai D, Schulze KM, Sothiratnam S, Hussain F, Shah BR, Pare G, Beyene J, Lear SA, for the South Asian Heart Risk Assessment Investigators. A Digital Health Intervention to Lower Cardiovascular Risk: A Randomized Clinical Trial. JAMA Cardiol 2016 Aug 1;1(5):601–606. doi: 10.1001/jamacardio.2016.1035

4. Johnston N, Bodegard J, Jerström S, Åkesson J, Brorsson H, Alfredsson J, Albertsson PA, Karlsson J-E, Varenhorst C. Effects of interactive patient smartphone support app on drug adherence and lifestyle changes in myocardial infarction patients: A randomized study. Am Heart J 2016 Aug 1;178:85–94. doi: 10.1016/j.ahj.2016.05.005

5. Redfern J, Santo K, Coorey G, Thakkar J, Hackett M, Thiagalingam A, Chow CK. Factors Influencing Engagement, Perceived Usefulness and Behavioral Mechanisms Associated with a Text Message Support Program. PLOS ONE Public Library of Science; 2016 Oct 14;11(10):e0163929. doi: 10.1371/journal.pone.0163929

6. Thakkar J, Redfern J, Thiagalingam A, Chow CK. Patterns, predictors and effects of texting intervention on physical activity in CHD – insights from the TEXT ME randomized clinical trial. Eur J Prev Cardiol 2016 Nov 1;23(17):1894–1902. doi: 10.1177/2047487316664190

7. Akhu-Zaheya LM, Shiyab WY. The effect of short message system (SMS) reminder on adherence to a healthy diet, medication, and cessation of smoking among adult patients with cardiovascular diseases. Int J Med Inf 2017 Feb 1;98:65–75. doi: 10.1016/j.ijmedinf.2016.12.003

8. Jones AR, Moser DK, Hatcher J. Using text messages to promote health in African-Americans: #HeartHealthyandCancerFree*. Ethn Health Taylor & Francis; 2018 Apr 3;23(3):307–320. PMID:27897049

9. Morawski K, Ghazinouri R, Krumme A, Lauffenburger JC, Lu Z, Durfee E, Oley L, Lee J, Mohta N, Haff N, Juusola JL, Choudhry NK. Association of a Smartphone Application With Medication Adherence and Blood Pressure Control: The MedISAFE-BP Randomized Clinical Trial. JAMA Intern Med 2018 Jun 1;178(6):802. doi: 10.1001/jamainternmed.2018.0447

10. Santo K, Hyun K, de Keizer L, Thiagalingam A, Hillis GS, Chalmers J, Redfern J, Chow CK. The effects of a lifestyle-focused text-messaging intervention on adherence to dietary guideline recommendations in patients with coronary heart disease: an analysis of the TEXT ME study. Int J Behav Nutr Phys Act 2018 May 23;15(1):45. doi: 10.1186/s12966-018-0677-1

11. Beratarrechea A, Abrahams‐Gessel S, Irazola V, Gutierrez L, Moyano D, Gaziano TA. Using mHealth Tools to Improve Access and Coverage of People With Public Health Insurance and High Cardiovascular Disease Risk in Argentina: A Pragmatic Cluster Randomized Trial. J Am Heart Assoc Wiley; 2019 Apr 16;8(8):e011799. doi: 10.1161/JAHA.118.011799

12. Brasier N, Raichle CJ, Dörr M, Becke A, Nohturfft V, Weber S, Bulacher F, Salomon L, Noah T, Birkemeyer R, Eckstein J. Detection of atrial fibrillation with a smartphone camera: first prospective, international, two-centre, clinical validation study (DETECT AF PRO). EP Eur 2019 Jan 1;21(1):41–47. doi: 10.1093/europace/euy176

13. Dorje T, Zhao G, Tso K, Wang J, Chen Y, Tsokey L, Tan B-K, Scheer A, Jacques A, Li Z, Wang R, Chow CK, Ge J, Maiorana A. Smartphone and social media-based cardiac rehabilitation and secondary prevention in China (SMART-CR/SP): a parallel-group, single-blind, randomised controlled trial. Lancet Digit Health Elsevier; 2019 Nov 1;1(7):e363–e374. PMID:33323210

14. Huo X, Krumholz HM, Bai X, Spatz ES, Ding Q, Horak P, Zhao W, Gong Q, Zhang H, Yan X, Sun Y, Liu J, Wu X, Guan W, Wang X, Li J, Li X, Spertus JA, Masoudi FA, Zheng X. Effects of Mobile Text Messaging on Glycemic Control in Patients With Coronary Heart Disease and Diabetes Mellitus. Circ Cardiovasc Qual Outcomes American Heart Association; 2019 Sep;12(9):e005805. doi: 10.1161/CIRCOUTCOMES.119.005805

15. Perez MV, Mahaffey KW, Hedlin H, Rumsfeld JS, Garcia A, Ferris T, Balasubramanian V, Russo AM, Rajmane A, Cheung L, Hung G, Lee J, Kowey P, Talati N, Nag D, Gummidipundi SE, Beatty A, Hills MT, Desai S, Granger CB, Desai M, Turakhia MP, Apple Heart Study Investigators. Large-Scale Assessment of a Smartwatch to Identify Atrial Fibrillation. N Engl J Med 2019 Nov 14;381(20):1909–1917. PMID:31722151

16. Yousuf H, Reintjens R, Slipszenko E, Blok S, Somsen GA, Tulevski II, Hofstra L. Effectiveness of web-based personalised e‑Coaching lifestyle interventions. Neth Heart J 2019 Jan 1;27(1):24–29. doi: 10.1007/s12471-018-1200-7

17. Zheng X, Spatz ES, Bai X, Huo X, Ding Q, Horak P, Wu X, Guan W, Chow CK, Yan X, Sun Y, Wang X, Zhang H, Liu J, Li J, Li X, Spertus JA, Masoudi FA, Krumholz HM. Effect of Text Messaging on Risk Factor Management in Patients With Coronary Heart Disease. Circ Cardiovasc Qual Outcomes American Heart Association; 2019 Apr;12(4):e005616. doi: 10.1161/CIRCOUTCOMES.119.005616

18. Broers ER, Widdershoven J, Denollet J, Lodder P, Kop WJ, Wetzels M, Ayoola I, Piera-Jimenez J, Habibović M. Personalized eHealth Program for Life-style Change: Results From the “Do Cardiac Health Advanced New Generated Ecosystem (Do CHANGE 2)” Randomized Controlled Trial. Psychosom Med 2020 May;82(4):409–419. doi: 10.1097/PSY.0000000000000802

19. Grau-Pellicer M, Lalanza J, Jovell-Fernández E, Capdevila L. Impact of mHealth technology on adherence to healthy PA after stroke: a randomized study. Top Stroke Rehabil Taylor & Francis; 2020 Jul 3;27(5):354–368. PMID:31790639

20. Redfern J, Coorey G, Mulley J, Scaria A, Neubeck L, Hafiz N, Pitt C, Weir K, Forbes J, Parker S, Bampi F, Coenen A, Enright G, Wong A, Nguyen T, Harris M, Zwar N, Chow CK, Rodgers A, Heeley E, Panaretto K, Lau A, Hayman N, Usherwood T, Peiris D. A digital health intervention for cardiovascular disease management in primary care (CONNECT) randomized controlled trial. Npj Digit Med Nature Publishing Group; 2020 Sep 10;3(1):1–9. doi: 10.1038/s41746-020-00325-z

21. Sengupta A, Beckie T, Dutta K, Dey A, Chellappan S. A Mobile Health Intervention System for Women With Coronary Heart Disease: Usability Study. JMIR Form Res 2020 Jun 3;4(6):e16420. doi: 10.2196/16420

22. Coorey G, Peiris D, Scaria A, Mulley J, Neubeck L, Hafiz N, Redfern J. An Internet-Based Intervention for Cardiovascular Disease Management Integrated With Primary Care Electronic Health Records: Mixed Methods Evaluation of Implementation Fidelity and User Engagement. J Med Internet Res 2021 Apr 26;23(4):e25333. doi: 10.2196/25333

23. Kang S-H, Baek H, Cho J, Kim S, Hwang H, Lee W, Park JJ, Yoon YE, Yoon C-H, Cho Y-S, Youn T-J, Cho G-Y, Chae I-H, Choi D-J, Yoo S, Suh J-W. Management of cardiovascular disease using an mHealth tool: a randomized clinical trial. Npj Digit Med Nature Publishing Group; 2021 Dec 3;4(1):1–7. doi: 10.1038/s41746-021-00535-z

24. Muralidharan S, Ranjani H, Anjana RM, Gupta Y, Ambekar S, Koppikar V, Jagannathan N, Jena S, Tandon N, Allender S, Mohan V. Change in cardiometabolic risk factors among Asian Indian adults recruited in a mHealth-based diabetes prevention trial. Digit Health SAGE Publications Ltd; 2021 Jan 1;7:20552076211039032. doi: 10.1177/20552076211039032

25. Feldman K, Duncan RG, Nguyen A, Cook-Wiens G, Elad Y, Nuckols T, Pevnick JM. Will Apple devices’ passive atrial fibrillation detection prevent strokes? Estimating the proportion of high-risk actionable patients with real-world user data. J Am Med Inform Assoc 2022 Jun 1;29(6):1040–1049. doi: 10.1093/jamia/ocac009

26. Sakakibara BM, Lear SA, Barr SI, Goldsmith CH, Schneeberg A, Silverberg ND, Yao J, Eng JJ. Telehealth coaching to improve self-management for secondary prevention after stroke: A randomized controlled trial of Stroke Coach. Int J Stroke SAGE Publications; 2022 Apr 1;17(4):455–464. doi: 10.1177/17474930211017699

27. Cheung NW, Redfern J, Thiagalingam A, Hng T-M, Marschner S, Haider R, Faruquie S, Huben AV, She S, McIntyre D, Cho J-G, Chow CK, Investigators TS. Effect of Mobile Phone Text Messaging Self-Management Support for Patients With Diabetes or Coronary Heart Disease in a Chronic Disease Management Program (SupportMe) on Blood Pressure: Pragmatic Randomized Controlled Trial. J Med Internet Res 2023 Jun 16;25(1):e38275. doi: 10.2196/38275

28. Li D-M, Lu X-Y, Yang P-F, Zheng J, Hu H-H, Zhou Y, Zhang L-J, Liu J-M. Coordinated Patient Care via Mobile Phone–Based Telemedicine in Secondary Stroke Prevention: A Propensity Score-Matched Cohort Study. J Nurs Care Qual 2023 Sep;38(3):E42. doi: 10.1097/NCQ.0000000000000693

29. Xu L, Tong Q, Zhang X, Yu T, Lian X, Yu T, Falter M, Scherrenberg M, Kaihara T, Kizilkilic SE, Kindermans H, Dendale P, Li F. Smartphone-based gamification intervention to increase physical activity participation among patients with coronary heart disease: A randomized controlled trial. J Telemed Telecare SAGE Publications; 2023 Feb 16;1357633X221150943. doi: 10.1177/1357633X221150943

30. Beckie TM, Sengupta A, Dey AK, Dutta K, Ji M, Chellappan S. A Mobile Health Behavior Change Intervention for Women With Coronary Heart Disease: A RANDOMIZED CONTROLLED PILOT STUDY. J Cardiopulm Rehabil Prev 2024 Jan;44(1):40. doi: 10.1097/HCR.0000000000000804
